# The Clinical Efficacy of Personalized Embryo Transfer Guided by the Endometrial Receptivity Array/Analysis on IVF/ICSI Outcomes: A Systematic Review and Meta-Analysis

**DOI:** 10.3389/fphys.2022.841437

**Published:** 2022-04-27

**Authors:** Zhenteng Liu, Xuemei Liu, Meimei Wang, Huishan Zhao, Shunzhi He, Shoucui Lai, Qinglan Qu, Xinrong Wang, Dongmei Zhao, Hongchu Bao

**Affiliations:** Department of Reproductive Medicine, Yantai Yuhuangding Hospital Affiliated to Qingdao University, Yantai, China

**Keywords:** endometrial receptivity array, personalized embryo transfer, repeated implantation failure, live birth rate, *in vitro* fertiization

## Abstract

**Objective:** To assess the prevalence of displaced window of implantation (WOI) in infertile women, and the clinical utility of personalized embryo transfer (pET) guided by the endometrial receptivity array/analysis (ERA) on IVF/ICSI outcomes.

**Methods:** The protocol was registered at Prospero: CRD42020204237. We systematically searched all published English literature related to the prevalence of WOI displacement and ongoing pregnancy rate/live birth rate in the overall good-prognosis infertile patients (GPP) and/or repeated implantation failure (RIF) patients undergoing IVF/ICSI-ET cycles after ERA test until August 2021.

**Result(s):** 11 published studies were enrolled in the final analysis. The estimate of the incidence of WOI displacement based on ERA was 38% (95%CI 19–57%) in GPP and 34% (95%CI 24–43%) in RIF, respectively. There was no difference in OPR/LBR between patients undergoing routine ET without ERA test and those who following pET with ERA (39.5 vs. 53.7%, OR 1.28, *p* = 0.49, 95%CI 0.92–1.77, *I* ^*2*^ = 0%) in relative GPP. Notably, the meta-analysis revealed that OPR/LBR of patients with RIF undergoing pET who had non-receptive ERA increased to the level of to those undergoing sET with receptive ERA (40.7 vs.49.6%, OR 0.94, *p* = 0.85, 95%CI 0.70–1.26, *I* ^*2*^ = 0%).

**Conclusion:** Considering the approximately one third of infertile women could suffered from displaced WOI, the ERA test emerged as a promising tool. Although the present meta-analysis demonstrates that patients with general good-prognosis may not benefit from ERA, pET guided by ERA significantly increases the chances of pregnancy for non-receptive patients with RIF of endometrial origin.

**Systematic Review Registration:** https://systematic.review.gov/, identifier [registration number]

## Introduction

Successful embryonic implantation requires two essential elements which includes a competent embryo and a receptive synchronized endometrium. Despite very effective advanced embryo selection tools such as preimplantation genetic testing for aneuploidy (PGT-A) or time-lapse imaging are now available and have achieved considerable improvements in *vitro* fertilization (IVF) outcomes (Munne, 2018; Rocafort et al., 2018), good-quality euploid embryos still fail to implant in about 1/3 of transfers (Forman et al., 2013). Implantation failure has remained as the primary drawback impeding IVF-ET along with ICSI (intracytoplasmic sperm injection) treatments, which has prompted further exploration into endometrial receptivity to assess whether this could aid to improve pregnancy outcomes.

The window of implantation (WOI) constitutes a short period in the menstrual cycle, where the endometrium acquires a functional status that supports blastocyst acceptance. The length of WOI is not consistent among all women, and some present WOI displacement (Galliano et al., 2015), which was traditionally monitored by ultrasound, histological, and molecular markers. Unfortunately, these methods of assessing the temporal boundaries of the physiologic WOI lack precision and objectivity (Lessey, 2011; He et al., 2021). In an estimated 30% of IVF cycles where transfer of embryo is conducted blindly, displacement of WOI happens and embryo-endometrial synchrony is not attained (Ruiz-Alonso et al., 2021).

The endometrial receptivity array/analysis (ERA) is the first commercial customized diagnostic approach that measures the expression of 248 endometrial genes, identifies the receptivity status (receptive or non-receptive) of an endometrium accurately, and determines the displacement of WOI (proliferative, pre-receptive and post-receptive) of a given patient in the clinical setting (Diaz-Gimeno et al., 2011; Diaz-Gimeno et al., 2013). Patients with receptive ERA would undergo standard embryo transfer (sET) in a subsequent cycle, while ones who had non-receptive (NR) results would been provided recommendations to adjust the ET timing, for what is termed to as a pET (personalized embryo transfer) (Ruiz-Alonso et al., 2013).

Several investigations have been carried out to explore the clinical utility of pET guided by the ERA on IVF/ICSI outcomes, and to discover the rate of WOI displacement in different infertile cohort, demonstrating contradictory or conflicting results. Because of skyrocketed utilization of the ERA and the high cost for conducting ERA (Ruiz-Alonso et al., 2021), the present meta-analysis aimed to determine if pET guided by the ERA improve pregnant outcomes in good-prognosis patient population (GPP, 0–2 prior failed ETs) and recurrent implantation failure (RIF)/poor-prognosis patient cohort separately. The secondary objective was to estimate the prevalence of displaced WOI in above two infertile population.

## Materials and Methods

### Protocol and Registration

The present research work was conducted as per PRISMA (Preferred Reporting Items for Systematic Reviews and Meta-Analyses) guidelines (Page et al., 2021). The protocol was registered at Prospero: CRD42020204237. No institutional review board approval was required for this report because it is a meta-analysis.

### Search Strategy

An electronic-centered screening search was done in Pubmed, Embase, Web of science, Cochrane Central Register of Controlled Trials, as well as Google Scholar, identifying all related published data until August 2021. The Medical Subject Headings (MeSH) along with key word terms utilized were “endometrial,” OR “endometrial receptivity array,” OR “endometrial receptivity analysis,” OR “ERA,” OR “personalized embryo transfer,” OR “personalized ET,” AND “ART,” OR “assisted reproductive techniques,” OR “ET,” OR “embryo transfer,” OR “IVF,” OR “*in vitro* fertilization,” OR “ICSI,” and “intracytoplasmic sperm injection.” We searched the reference section of the enrolled articles, relevant reviews along with meta-analyses to identify other relevant articles. The screening was limited to English publication language.

### Inclusion and Exclusion Criteria

Study designs: Experimental, observational and randomized controlled trials (RCT), excluding review articles or case reports.

Population: Infertile patients undergoing IVF/ICSI-ET cycles (with fresh or frozen D3 embryos or D5-6 blastocysts) after pET based upon ERA test.

#### Intervention: ERA/pET

Method of intervention: Endometrial receptivity was explored *via* ERA in collected biopsies during WOI (7 days post the spontaneous surge of the luteinizing hormone in natural cycles, i.e., LH+7; 5 days post administration with P during hormone replacement treatment cycles, i.e., P+5). Individuals with a receptive ERA result underwent a sET in the following cycle *via* the same approach as well as on the exact cycle day as their original ERA. Individuals with a non-receptive ERA outcome selected either to receive an additional mock cycle and a repeat ERA assessment on the altered day suggested *via* their ERA outcome, or to continue with pET on the suggested day without an additional confirmatory biopsy.

Comparator: The overall good-prognosis infertile patients (GPP, 0–2 failed ETs) pursuing ERA or not receiving the ERA test. Repeated implantation failure (RIF) patients undergoing pET or standard ET (sET) according to the ERA results.

#### Outcomes:

Primary outcome: Prevalence of displaced WOI; OPR/LBR (Ongoing pregnancy/live birth rate).

Secondary outcome: PR/CPR (Pregnancy/clinical pregnancy rate); IR (Implantation rate); MR (Miscarriage rate).

#### Outcome Definition:

OPR/LBR: “Ongoing pregnancy” computed *via* accounting for the viable intrauterine pregnancy proceeding past 12 weeks of gestation. ‘‘Live birth’’ constituted the delivery of one or more live infant(s) post 24 weeks’ gestation.

PR/CPR: “PR” defined as total number of hCG (beta-human chorionic gonadotropin)-positive (value > 10 IU/L) patients stratified via the overall ET number. “CPR” defined as the visualization *via* ultrasonography of one or more intra-uterine gestational sacs harboring at least one embryo with heartbeat.

IR: computed *via* dividing the number of intra-uterine gestational sacs seen on ultrasound *via* the ET number.

MR: constituted fetal loss prior to the 20th week of gestation.

### Study Selection and Data Extraction

Screening of the articles’ titles along with abstracts was done by three independent researchers (ZL, MW, and HZ.). After that, full text-screening was done to determine relevant articles as per the inclusion along with the exclusion criteria. Any discrepancies were settled by a fourth researcher (X.M.L.). Data abstraction was done by three independent researchers (SH, S,L and QQ). One author (H.C.B) supervised the selection along with the data abstraction process. Comparison of the results was done and any discrepancies discussed and settled *via* consensus.

### Risk of Bias

Assessment of the enrolled articles’ methodological quality was done by three independent researchers (Z.T.L, X.M.L, and M.M.W) *via* the “Modified Newcastle-Ottawa scoring items (Table 1)” (Stang, 2010). Five distinct domains were used to assess the articles’ quality “sample representativeness,” “Ascertainment of Non-receptive diagnosis according to ERA,” “sampling technique,” “quality of description of the population,” and “incomplete outcome data” (Table 1). On the basis of the overall assigned points, articles were assigned to low bias risk (>3 points) or high bias risk (<3 points). Any disagreements regarding researcher’s judgements were settled by a fourth reviewer (H.C.B) *via* consensus.

**TABLE 1:** Modified Newcastle-Ottawa scoring items.

(1) Sample representativeness:
1 point: Sample size was greater than or equal to 100 participants and exclusion rate was lower than 20%.
0 points: Sample size was less than 100 participants or exclusion rate was higher than 20%
(2) Sampling technique:
1 point: Patients recruited consecutively or randomly (randomization criteria clarified)
0 points: Potential convenience sampling or unspecified sampling technique.
(3) Ascertainment of displaced WOI or non-receptive (NR) diagnosis:
1 point: The study employed a customized ERA array (containing 238 genes expressed at the different stages of the endometrial cycle and is coupled to a computational predictor that is able to identify the receptivity status of an endometrial sample and diagnose the displaced WOI (dWOI) of a given patient regardless of the sample's histologic appearance)
0 points: The study employed histological dating or other techniques to diagnose the endometrial status, or no precise/invalid timing for endometrial biopsy
(4) Quality of description of the population:
1 point: The study reported a clear description of the population (e.g. age, kind of reproductive disorder, diagnostic criteria for the reproductive disorder) with proper measures of dispersion (e.g., mean, standard deviation)
0 points: The study did not report a clear description of the population, incompletely reported descriptive statistics, or did not report measures of dispersion
(5) Incomplete outcome data:
1 point: The study reported complete data about implantation rate, ongoing pregnancy/live birth rate, miscarriage rate.
0 points: Selective data reporting cannot be excluded

The individual components listed above are summed to generate a total modified Newcastle-Ottawa risk of bias score for each study. Total scores range from 0 to 5. For the total score grouping, studies were judged to be of low risk of bias (≥3 points) or high risk of bias (<3 points).

### Statistical Analysis

Stata software version 13.0 was used to evaluate the prevalence of displaced WOI in infertile women. Quantitative synthesis and subgroup analyses were independently conducted via the Review Manager version 5.3. by two authors (X.R.W and D.M.Z). Comparisons of all results were done, and any differences discussed. Study outcomes are given as odds ratio (OR) with 95% confidence interval (95% CI), with P =<0.05 signifying statistical significance. If heterogeneity with significance existed (*I* ^*2*^ ≥ 50%), we adopted a random-effects approach; otherwise, a fixed-effects model was applied. Sensitivity assessments were also carried out via estimation of the combined prevalence in the absence of each article to determine the impact of every article on the pooled prevalence by Stata software (SL and QQ).

## Result

### Studies Included for Meta-Analysis

**FIGURE 1:**
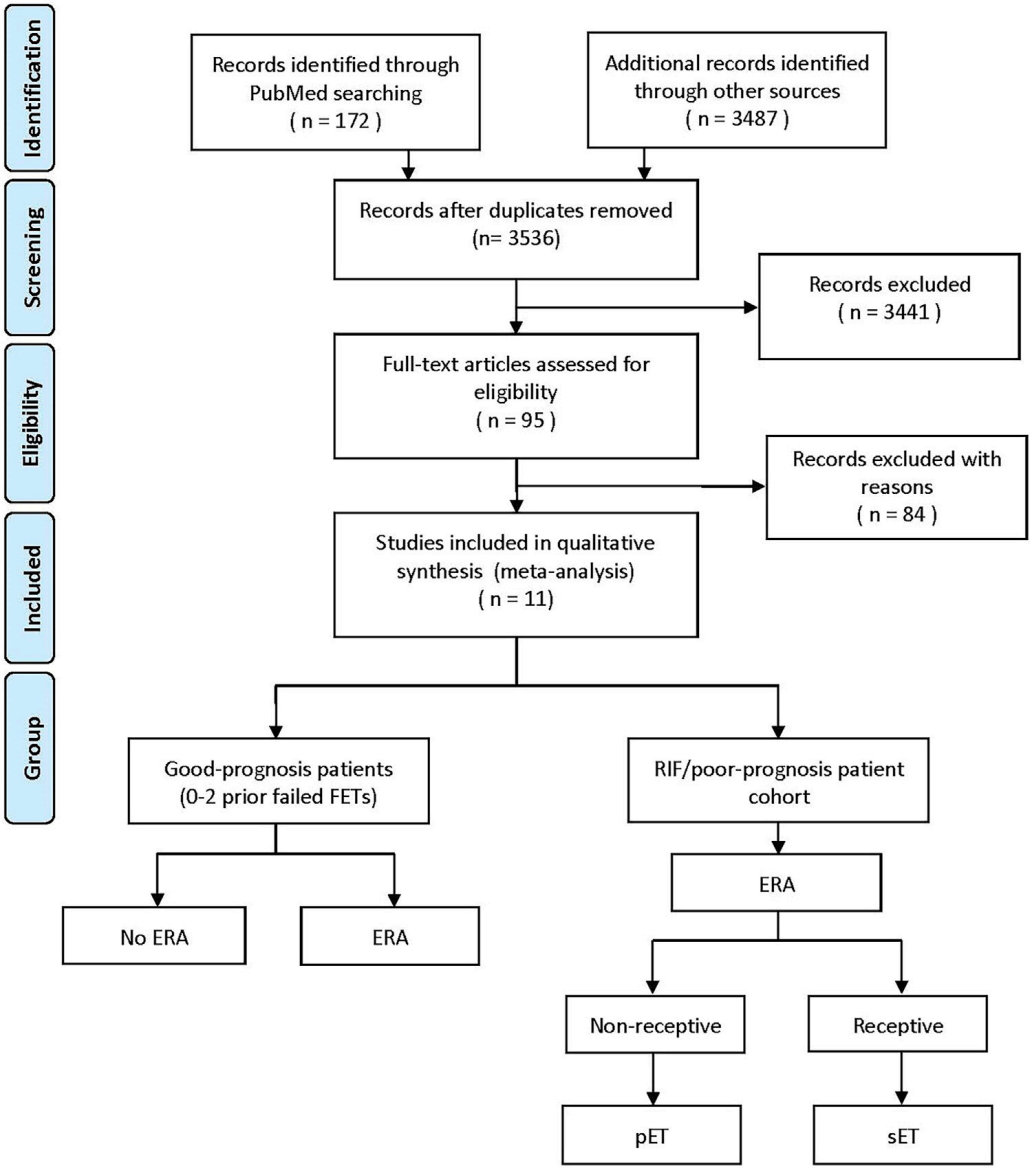
PRISMA (Preferred Reporting Items for Systematic Reviews and Meta-Analyses) flowchart.

After searching in target databases and screening titles, abstracts or manuscripts, 11 articles were finally included in this meta-analysis (Figure 1). The characteristics of the literatures chosen for quantitative synthesis are listed, summarized, and compared in Table 2. Most studies were observational, including two prospective (Ruiz-Alonso et al., 2013; Riestenberg et al., 2021) and egiht retrospective (Mahajan, 2015; Hashimoto et al., 2017; Bassil et al., 2018; Tan et al., 2018; Neves et al., 2019; Patel et al., 2019; Cohen et al., 2020; Cozzolino et al., 2020), and only one was multicenter RCT (Simon et al., 2020). Ruiz-Alonso et al. (2013), Mahajan et al. (2015) and Tan et al. (2018) reported two different cohorts (RIF and GPP [0–2 previous ET failed] patients) in their studies. In Neves *et al.* study (Neves et al., 2019), the subjects included patients with≥1 previous failed Euploid-ET or ≥2 failed Donor-ET. Since a high number of earlier transferred euploid embryo (1.75 ± 0.85) and failed Donor-ET cycles (2.87 ± 0.96), we attribute this study to RIF/poor-prognosis patient cohort.

**TABLE 2:** General features of the 11 included articles.

Authors and year	Study Design	Country, and Time of realization	Participants and Main Inclusion Criteria	Endometrial sampling and processing	Age (Cases/Controls) (year)	Endometrium Preparation Protocol for ET and embryo Type and Number	Outcomes
1. Ruiz-Alonso et al., 2013	Multicentre prospective cohort study	Spain; 20 months during 2011–2012	85 patients with RIF who underwent ≥3 previous failed IVF-ET cycles or 4 high-quality embryo transferred;	Endometrial biopsies were collected from the uterine fundus with the use of Pipelle catheters from Cornier Devices or similar, under sterile conditions either on day LH+7 in a natural cycle or on day P+5 in an HRT cycle	38.4 ± 4.7/39.9 ± 5.1	Nature/HRT; Cleavage stage embryo or blastocysts, and number was not mentioned	PR; IR; MR
			25 patients in the control group with the same age inclusion criteria within the same time period as the RIF patients included in this study but who had only one or no previous failed cycles				
			Normal ovarian reserve				
			Normal karyotypes				
			Negative testing for antiphospholipid antibodies				
			Normal uterine cavity				
2. Mahajan 2015	Monocentric retrospective study	India	80 patients with RIF was defined as ≥ 2 failed IVF-ET cycles; 93 patients with one IVF failed	Endometrial biopsies were collected from the uterine cavity with the use of Pipelle catheters	34.8 ± 4.8/33.3 ± 4.0	HRT	PR; OPR; IR
			Normal ovarian reserve	on day P + 5 in an HRT cycle		2 good quality blastocysts	
			Normal karyotypes				
			Negative testing for antiphospholipid antibodies				
			Normal uterine cavity				
3. Hashimoto et al., 2017	Two-centers retrospective study	Japan 2014 - July 2017	50 patients with RIF and a past history of repeated implantation failure with ≥3 good-quality embryo transfers	The endometrial biopsy was performed from the uterine fundus by using a catheter called “ENDOSUCTION” either on day P+5 in the HRT cycles or on day hCG+7 or LH+7 in the natural cycles	38.42 ± 3.4/40.08 ± 5.16	Nature/HRT	PR, Take-home baby rate; IR; MR
			Normal uterine cavity by ultrasound test (hysteroscopy in necessary)			Only 1 blastocyst	
4. Tan et al., 2018	Monocentric retrospective study	Canada	62 patients with RIF defined as ≥ 2 prior failed fresh or frozen embryo transfer cycles. 26 patients with 0–1 IVF failed	The endometrial biopsy was performed with a Pipelle catheter after five full days of progesterone administration (P + 5) in the HRT cycles	37.5 ± 4.8	HRT; frozen blastocysts or euploid embryos, and number was not mentioned	IR, LBR and OPR
		October 2014 - July 2017					
5. Bassil R. et al., 2018	Single-center retrospective cohort study	Canada	53 consecutive good-prognosis patients (0–2 previous frozen embryo transfers) receiving ERA test; 503 patients (control group) underwent their first or second FET cycles without performing the ERA testing during the same period of time	The endometrial biopsy was performed on day P + 5 in the HRT cycles or on day LH + 7 in the natural cycle	36.3 ± 0.4/35.6 ± 4	Modified nature cycles/HRT; Frozen day-5 blastocyst, and number was not mentioned	OPR
		April 2016 - March 2017					
6. Patel JA. et al., 2019	Monocentric retrospective study	India	248 RIF women having ≥3 unsuccessful fresh and/or frozen embryo transfer cycles each with one or two morphologically high-grade embryos using self or donor oocytes in which no cause for RIF was found after thorough infertility workup	The endometrial biopsy was collected from the uterine fundus with the use of Pipelle catheters on P + 5 days	33.67 ± 5.12/34.11 ± 4.49	HRT	PR; CPR; OPR; IR; MR
		July 2013 - September 2017	Normal karyotypes			Cleavage stage embryo, blastocysts, or ovum donation and number was not mentioned	
			Normal uterine cavity				
7. Neves AR. et al., 2019	Single-centerretrospective cohort study	Spain	24 patients with≥1 previous failed Euploid-ET or 32 patients≥2 failed Donor-ET who underwent an ERA test. Controls were patients with ≥1 previously failed Euploid-ET (*n* = 119) or ≥2 failed Donor-ET (*n* = 158) without performing an ERA test.	Endometrial biopsy was performed on day P + 5 using a Pipelle^®^ endometrial sampler (Laboratoire CCD, Paris, France) or similar device, under sterile conditions	39.25 ± 3.99/39.18 ± 3.80; 42.19 ± 3.34/43.40 ± 4.13	HRT	IR, CPR
		October 2012-December 2018	Normal karyotypes			Blastocysts/PGT-A/donation, and number was not mentioned	
			Normal thyroid function				
			No condition interfering with immune system				
			No uterine malformation				
8. Simon. et al., 2020	Multicentre randomized controlled trial	Europe, United States of America and Asia	Women scheduled for their first blastocyst transfer were included. Inclusion criteria were age 37 years or younger, BMI of 18.5–30 and normal ovarian reserve (antral follicle count ≥8 and FSH <8 IU/ml). The intention to treat (ITT) analysis was conducted in 434 patients, pET (n = 141), FET (*n* = 148) or fresh embryo transfer (*n* = 145). Per protocol analysis was conducted in 266 patients (pET [*n* = 80], FET [*n* = 92] or fresh embryo transfer [*n* = 94]). We chose data from per protocol analysis for meta-analysis.	Endometrial biopsies were collected from the uterine fundus using a Pipelle catheter from Cornier^®^ devices (CCD Laboratories, Paris, France) or similar, under sterile conditions on day P + 5 in an HRT cycle	33 ± 3.1/32.8 ± 3.4/32.7 ± 3.3	HRT/fresh ET Blastocyst stage (day 5 or 6)	LBR, CLBR, PR, IR, CMR
		November 2013-April 2017ycle					
9. Cozzolino,.et al., 2020	Multicenter retrospective cohort study	Data from IVIRMA clinics in Europe; 2013–2018	93 moderate RIF patients (≥3 failed good-quality embryos in different single fresh or frozen, own or donated embryo transfers) between 18 and 45 years old	Endometrial biopsies were collected from the uterine fundus on day P + 5 in the HRT cycles or on day LH + 7 in the natural cycles, and samples were	38.5–38.6/37.9–38.2	Nature/HRT Blastocysts/PGT-A/donor, and number was not mentioned	IR, OPR
			No uterine malformation				
			analyzed by iGenomix according to their protocol				
			Blastocysts/PGT-A/donor, and number was not mentioned				
10.Cohen, et al., 2020	Single-centerretrospective cohort study	Canada; May 2014-March 2019	97 RIF women having ≥2 failed consecutive embryo transfers with morphologically high-quality blastocysts	An endometrial biopsy was performed on day P + 5 in the HRT cycle An ‘ENDOCELL’ pipelle (Wallach Surgical Devices, United States of America) was used to perform the endometrial biopsy in standard aseptic fashion	36.1 ± 4.0/35.9 ± 3.8	HRT; Frozen blastocyst, and number was not mentioned	CPR, LBR, IR, MR
			No uterine malformation				
11. Riestenberg, C.et al., 2021	Monocentric prospective cohort study	United States of America; January 2018-April 2019	228 patients underwent their first single euploid programmed FET during the study period. Of those, 147 were ERA/pET cycles, and 81 were standard ET cycles without ERA. Natural cycle and minimalstimulation	Biopsy was performed with a suction pipelle on P+5 in HRT, and the ERA was performed using Igenomix	34.9 ± 3.8/36.9 ± 3.8	HRT; Autologous single euploid blastocyst	CPR, LBR, Biochemical PB, MR
			FET cycles were excluded				

ERA, endometrial receptivity array/analysis; RIF, recurrent implantation failure; FET, frozen embryo transfer; pET, personalized embryo transfer; HRT, hormone replacement therapy; PGT-A, preimplantation genetic testing for aneuploidy; P, progesterone; LH, luteinizing hormone; PR, pregnancy rate; CPR, clinical pregnancy rate; OPR, ongoing pregnancy rate; IR, implantation rate; MR, miscarriage rate; LBR, live birth rate.

In general, the method and timing of endometrial biopsy for ERA (IGENOMIX, Valencia, Spain) were consistent, performing from the uterine fundus *via* a Pipelle catheter either on day P+5 in the HRT cycles or on day hCG+7 or LH+7 in the natural cycles. Consistent with the evaluation of risk of study bias, all articles were rated as low risk (Table 3).

**TABLE 3:** Authors’ judgement of study quality according to the “Modified Newcastle-Ottawa Risk of Bias Scoring System.ˮ

Authors and year	Sample representativeness	Sampling technique	Ascertainment of non-receptive Diagnosis	Quality of Description of the Population	Incomplete Outcome data	Total score	Risk of bias
Ruiz-Alonso et al. (2013)	★	**—**	★	**—**	★	★★★	low
Mahajan. (2015)	★	**—**	★	★	**—**	★★★	low
Hashimoto et al. (2017)	**—**	**—**	★	★	★	★★★	low
Tan et al. (2018)	**—**	**—**	★	★	★	★★★	low
Bassil R.et al., 2018	★	★	★	★	**—**	★★★★	low
Patel et al. (2019)	★	**—**	★	**—**	★	★★★	low
Neves et al. (2019)	★	**—**	★	★	**—**	★★★	low
Simon et al. (2020)	**—**	★	★	★	★	★★★★	low
Cozzolino et al. (2020)	★	**—**	★	★	**—**	★★★	low
Cohen et al. (2020)	**—**	**—**	★	★	★	★★★	low
Riestenberg et al. (2021)	★	**—**	★	★	★	★★★★	low

### The Rate of WOI Displacement

**FIGURE 2:**
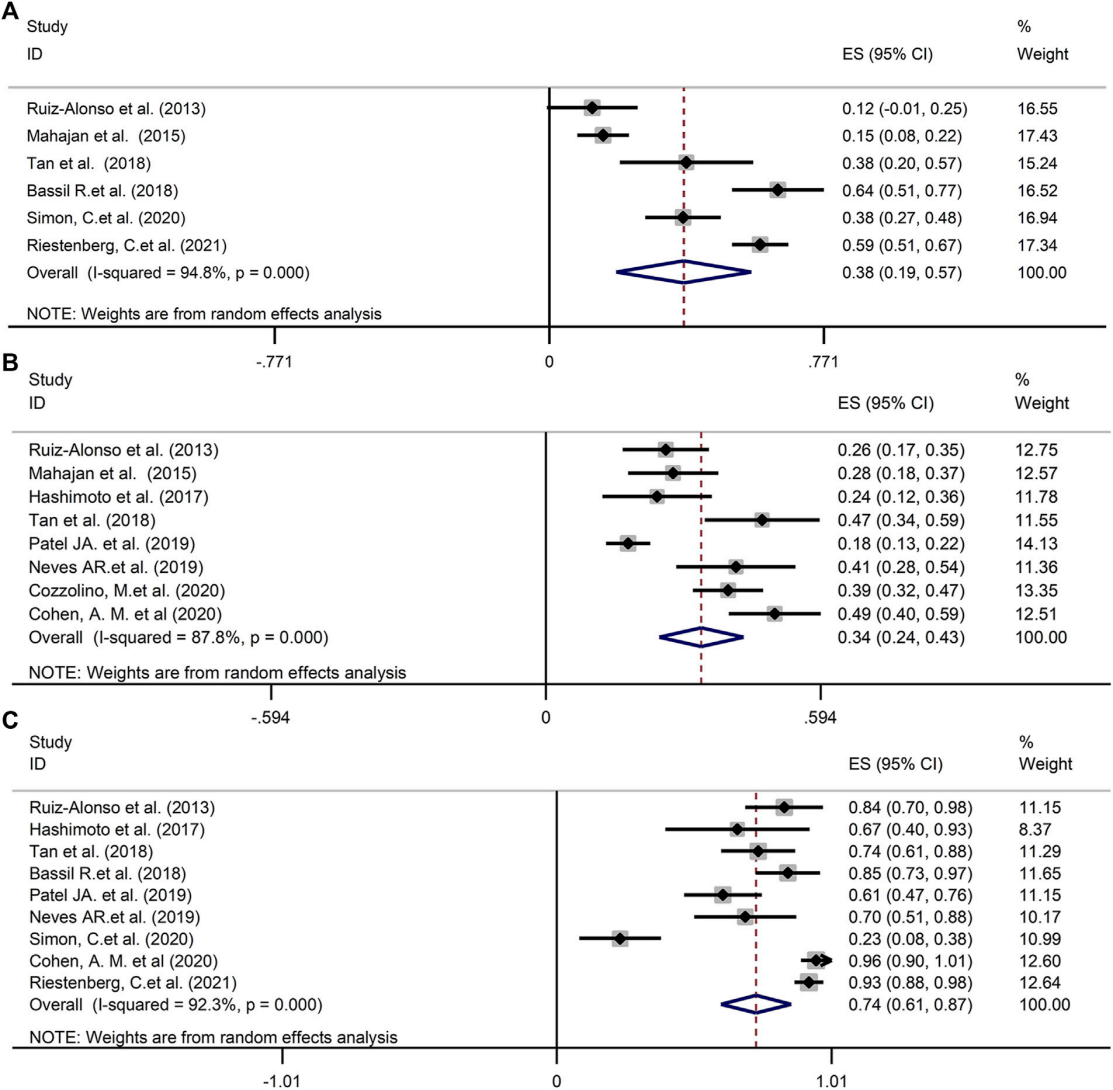
**(A)** The prevalence of WOI displacement in good-prognosis population. **(B)** The prevalence of WOI displacement in RIF/poor-prognosis patient cohort. **(C)** The proportion of the pre-receptive endometrium in the non-receptive ERA status. WOI, Window of implantation; ERA, Endometrial receptivity array/analysis; RIF, Recurrent implantation failure.

The estimate of the incidence of WOI displacement based on ERA was 38% (95%CI 19–57%, *n* = 6 trials) in good-prognosis patient population and 34% (95%CI 24–43%, *n* = 8 trials) in RIF/poor-prognosis patient cohort, respectively (Figures 2A,B). The rate of displaced WOI followed a normal distribution with a random effect model showing high heterogeneity (*I* ^*2*^ = 95.9 and 87.9%, *p* < 0.001) in above two cohort, as shown in Figures 2A,B. A further analysis was generated to assess the proportion of the pre-receptive endometrium (i.e., had not reached the WOI yet) in the non-receptive ERA status. As described in Figure 2C, the prevalence of pre-receptive status was 74% (95%CI 61–87%, *I* ^*2*^ = 92.3%, *n* = 9 trials).

**FIGURE 3:**
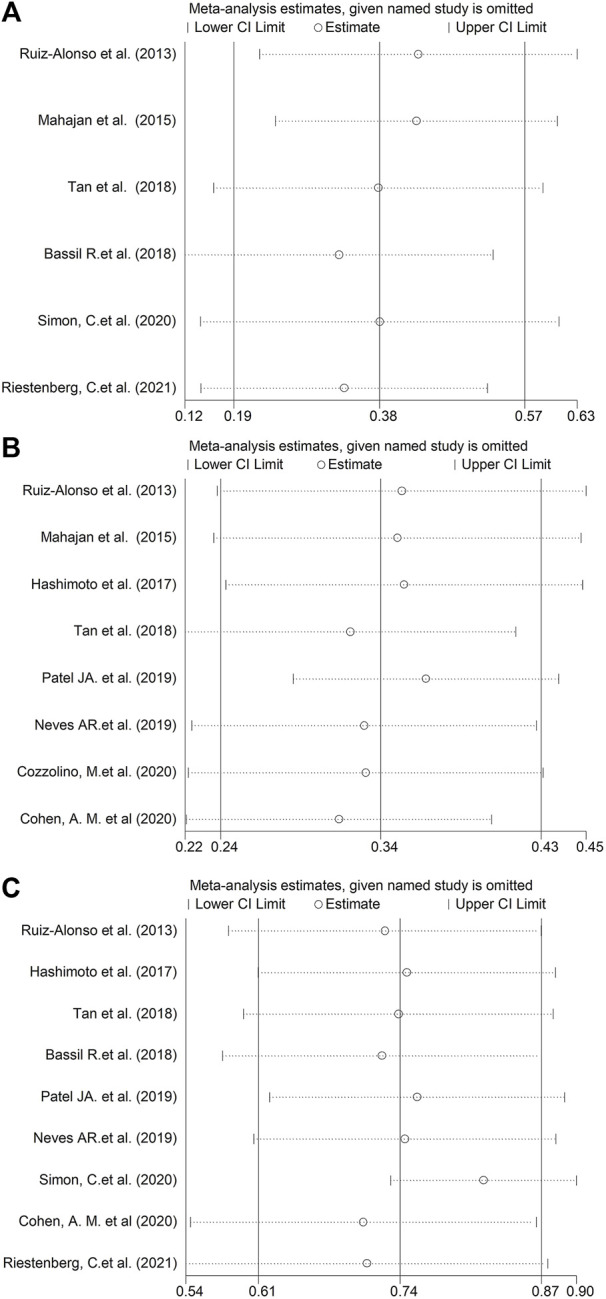
Sensitivity analysis, generated by estimating the combined proportion in the absence of each study. **(A)** GPP. **(B)** RIF. **(C)** Pre-receptive NR.

A sensitivity assessment was conducted by estimating the combined prevalence in the absence of each study, in order to assess its influence (Figure 3). As depicted in Figure 3, when we exclude Bassil R.et al. in GPP, Patel JA. et al. in RIF or Simon, C.et al. in pre-receptive NR status, the change of the incidences was relative obvious in above three groups.

### Synthesis of Clinical Results

#### ERA vs. No-ERA in GPP

**FIGURE 4:**
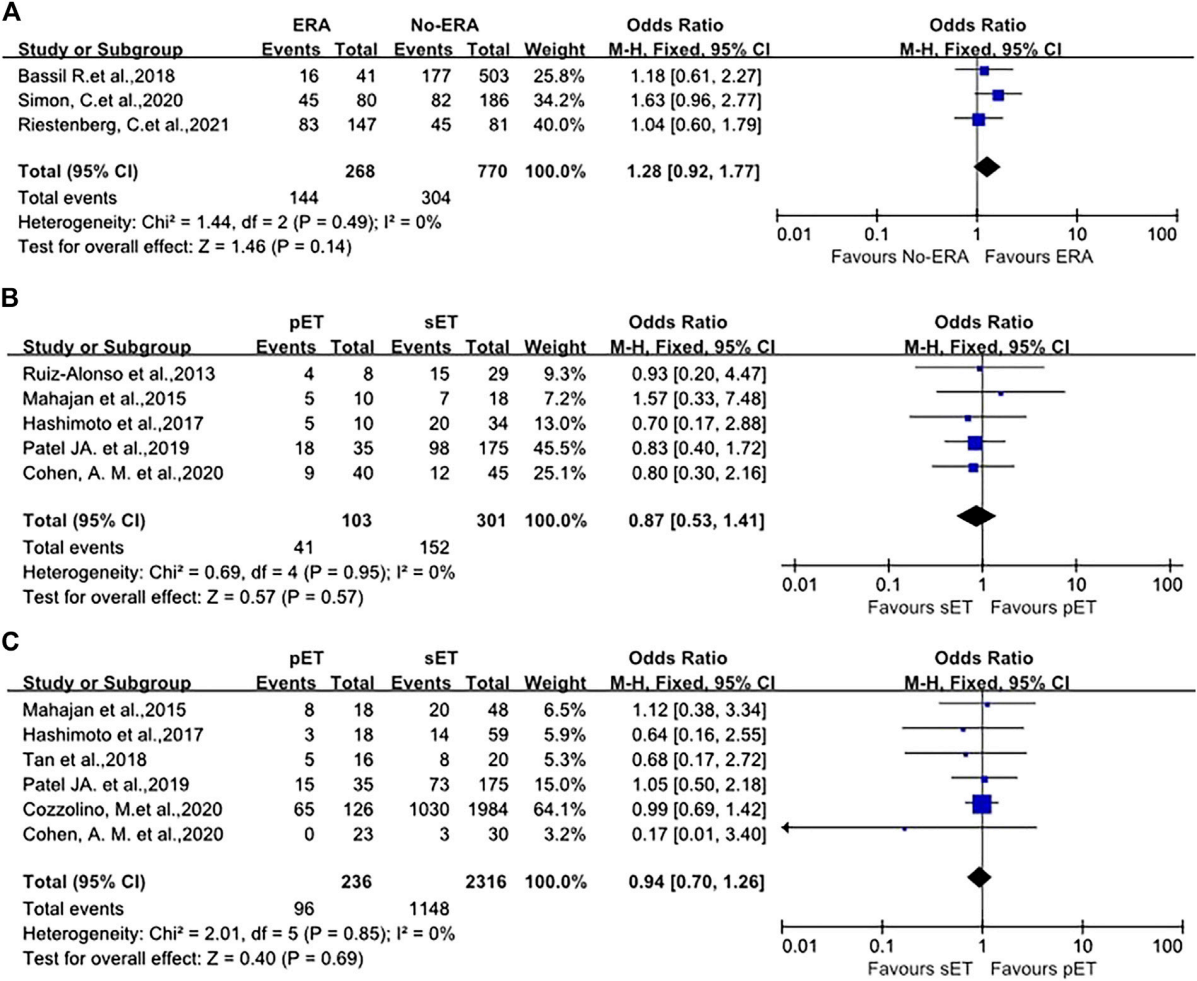
**(A)** Forest plot of ongoing pregnancy/live birth rate in good-prognosis population with ERA or without ERA. **(B)** Forest plot of the pregnancy/clinical pregnancy rate in RIF patients with pET or sET guided by ERA. **(C)** Forest plot of ongoing pregnancy/live birth rate in RIF patients with pET or sET guided by ERA. ERA, endometrial receptivity array/analysis; RIF, recurrent implantation failure; pET, personalized embryo transfer; sET, standard embryo transfer.

No remarkable difference was seen in OPR/LBR between patients undergoing ET with routine timing without ERA test and those who received endometrial biopsy with ERA following pET (39.5 vs. 53.7%, OR 1.28, *p* = 0.49, 95%CI 0.92–1.77, *I* ^*2*^ = 0%, Figure 4A) in good-prognosis patient population, irrespective of if the progesterone duration was changed on the basis of ERA results. Sensitivity assessment was not performed because of minimal inconsistency (*I* ^*2*^ = 0%).

#### pET vs. sET Guided by ERA in RIF

The pregnancy/clinical pregnancy rate (PR/CPR) of 404 participants in five studies were meta-analyzed. The data illustrated that the PR/CPR of patients with RIF possessing NR ERA and following pET was comparable to those who had receptive ERA who underwent standard ET (OR 0.87, *p* = 0.95, 95%CI 0.53–1.41, *I* ^*2*^ = 0%, Figure 4B). Specifically, the PR/CPR was 39.8% (41/103) in pET group versus 50.5% (152/301) in sET group.

OPR/LBR was reported in six studies, with a total of 2552 ET cycles. The meta-analysis revealed that OPR/LBR after pET in NR ERA cases were similar to that after sET in receptive ERA RIF patients (OR 0.94, 95%CI 0.70–1.26, *I* ^*2*^ = 0%, *p* = 0.85, Figure 4C). The OPR/LBR in pET group was 40.7% (96/236) versus 49.6% (1148/2316) in sET group.

**FIGURE 5:**
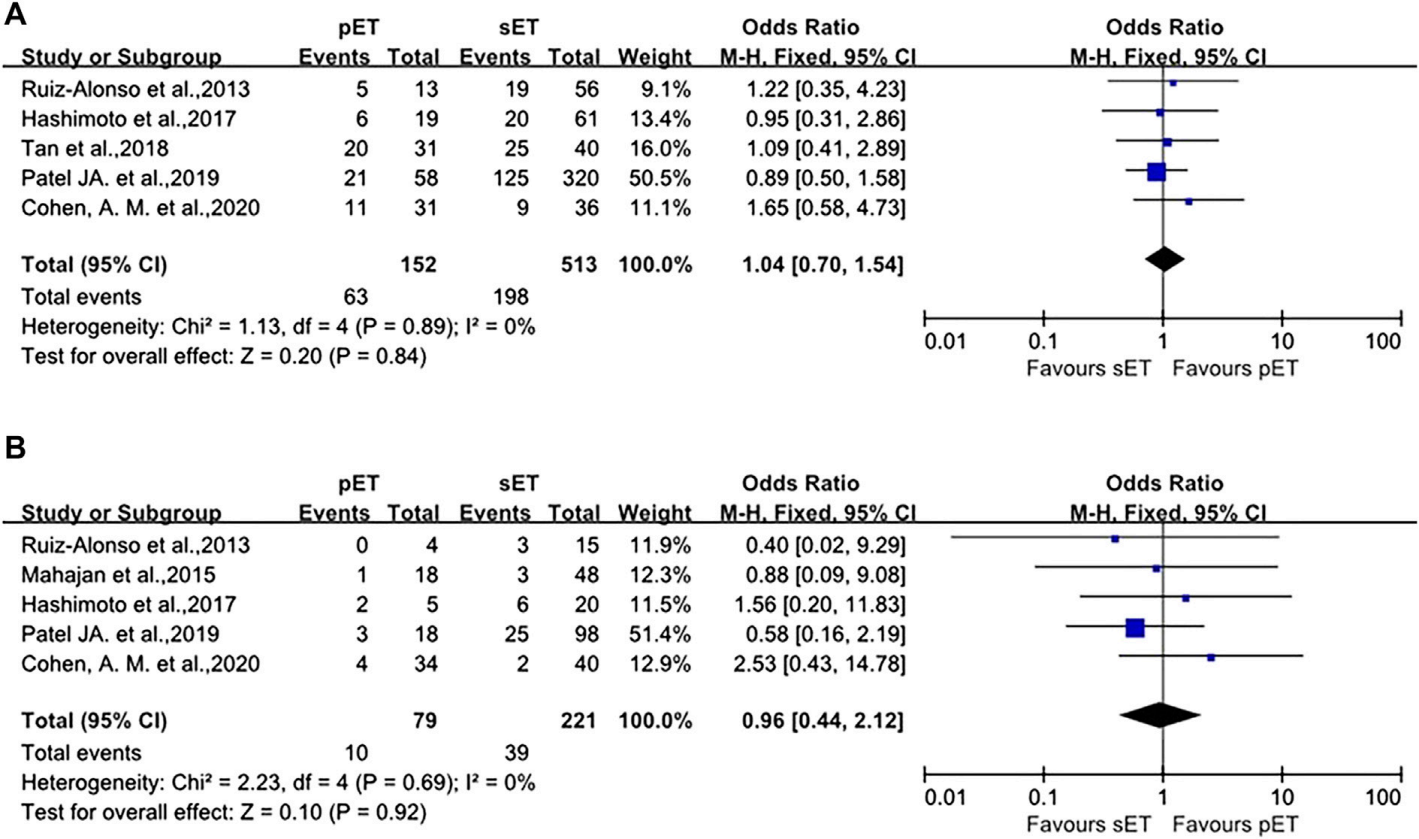
pET vs. sET guided by ERA in RIF patients. **(A)** implantation rate. **(B)** miscarriage rate. ERA, endometrial receptivity array/analysis; RIF, recurrent implantation failure; pET, personalized embryo transfer; sET, standard embryo transfer.

In terms of IR and MR, there were also no differences between the groups (OR 1.04, 95%CI 0.70–1.54, *p* = 0.89; OR 0.96, 95%CI 0.44–2.12, *p* = 0.69, as shown in Figure 5). Sensitivity analysis was not carried out because of minimal inconsistency (*I* ^*2*^ = 0%) in above comparisons.

## Discussion

Although great promise was brought by commercially available ERA to personalized medicine outcome and numerous investigations have been conducted in these areas, incongruent or even contradictory data are frequently reported. To the best of our knowledge, this is the first meta-analysis to evaluate the efficacy of ERA/pET on IVF/ICSI outcomes.

### Prevalence of Displaced WOI

After analysis of 11 studies, the pooled prevalence of displaced WOI according to ERA in good-prognosis patients and RIF cohort was estimated to be 38 and 34% separately. The fraction of receptive *vs*. non-receptive ERA results in above infertile patients has differed remarkably in different studies (12%–64%, 18%–49%, Figures 2A,B). A limitation regarding cross-study comparison includes heterogeneity of the population of patients in different literatures. Moreover, because there is no consensus in RIF definition, each investigation selected its own definition of the condition (≥2 or ≥3 failed ETs, as shown in Table 2). Due to the huge heterogeneity, we conducted sensitivity assessment to explore the combined prevalence in the absence of every study (Figure 3). The data illustrated that the absence of either of the three studies (Bassil et al., 2018; Patel et al., 2019; Simon et al., 2020) markedly changed the overall prevalence, hence exhibiting the significance of cohort variation among the included studies.

WOI period differs among all women, and some exhibit displacement of WOI, which could delay, narrow, or advance the WOI(Galliano et al., 2015). This might result in embryo-endometrial asynchrony, which often leads to failure in implantation or even RIF(Teh et al., 2016). It is worth noting that the present results indicate high prevalence (one third) of displaced WOI in infertile population; thus, when the etiology of infertility or repeated implant failure was searched, we cannot ignore the possibility of abnormal endometrial WOI, so that patients can receive the individualized treatment to attain pregnancy earlier.

Surprisingly, the meta demonstrated good-prognosis patients has a slightly greater risk of displaced WOI than RIF. Numerous factors impact endometrial gene expression including race, type of trigger employed in final oocyte maturation, endometrium preparation protocol, and body mass index (BMI) (Bermejo et al., 2014; Comstock et al., 2017). Thus, better comprehension of the impact of different factors on ERA outcomes would be valuable.

In terms of the classification of non-receptive ERA, the estimated prevalence of pre-receptive status was 74% in our present meta. The only one study reported that pre-receptive status accounted for a smaller proportion (7/30, 23%) in NR endometrial samples (Simon et al., 2020), but other articles all showed that majority of NR ERA patients were pre-receptive, which may be linked to race, small sample size and regional cohort in that study. This phenomenon certainly warrants further mechanistic investigation.

### ERA *vs.* No-ERA in GPP

The present review included 1,038 good-prognosis women (from three studies) (Bassil et al., 2018; Simon et al., 2020; Riestenberg et al., 2021) undergoing blastocysts transfer cycle. Patients in the intervention group (*n* = 268) received the appropriate adjustment in timing of FET according to the ERA result, whereas controls (*n* = 770) were standard timing ET cycles without ERA test. No remarkable difference was reported between groups regarding the primary outcome OPR/LBR (39.5 vs. 53.7%, OR 1.28, 95%CI 0.92–1.77, *I* ^*2*^ = 0%, *p* = 0.49, Figure 4A). As such, good-prognosis patients (0–2 previous failed ETs) may not benefit from ERA/pET. Our results indicate that ERA as a prognostic indicator may not be effective, as well as the utilization of pET based on ERA in overall good-prognosis population.

Implantation failure may be caused by WOI displacement and/or its disruption *via* molecular pathologies not linked to timing (Sebastian-Leon et al., 2018). Displacement (asynchrony) and disruption (pathology) may present independently or together in the same patient (Valdes et al., 2017). Infertile patients with displacement of WOI could benefit from ERA/pET, whilst individuals with disrupted WOI should be identified and further research undertaken for design of new treatments.

### pET *vs.* sET Guided by ERA in RIF

Unexplained RIF is a remarkable issue of infertility which remains fully unexplored, and it is extraordinarily needed to distinguish etiology to optimize the success rate of these patients. Notably, the clinical influence of pET in individuals with NR RIF was enhanced by the present meta where PR/CPR and OPR/LBR escalated to the extent of receptive RIF individuals.

Not all RIF with displaced endometrial WOI is pathology (disruption) however our failure to diagnose, as well as predict the correct time window with receptive endometrium in the past (Kliman and Frankfurter, 2019). In other words, some harbor different timing for receptivity of the endometrium, and individualized timing for transfer of the embryo could be beneficial in such individuals. Individualized treatment is a well-accepted concept in human reproduction, from the kind and dosage of gonadotropin in COH on the basis of ovarian reserve along with BMI, and determination of the fertilization approach (ICSI, IVF, or both) as per the sperm characteristics and clinical setting, to the criteria of development of the embryo based on the number, as well as quality of available embryos. It is intriguing that the status of the endometrium in all patients is often treated the same at the time of ET, which is only based on the stage of embryo development and is adjusted *via* administering P/hCG in the luteal phase (Patel et al., 2019). Gladly, ERA test is new, accurate, as well as sensitive in identification of genetic expressions in the endometrium to determine embryo transfer timing (Ruiz-Alonso et al., 2013). Results on receptive ERA exhibit a potential peak endometrial receptive window for a high-quality blastocyst to implant. In light of our data, pET guided by ERA considerably increases the chances of pregnancy for non-receptive patients with unexplained RIF. This showed that normal pregnancy along with implantation rates might be attained *via* pET in individuals with RIF of endometrial origin if synchrony between the embryo and receptive endometrium is accomplished.

Notably, we also found that the general OPR/LBR in GPP (with and without ERA) is 43.2%, but in RIF with pET guided by ERA seems to be higher, i.e., 48.7%. First, there was only one study which transferred single euploid embryo with 40% weight in GPP population (Riestenberg et al., 2021), but there were two studies which transferred euploid embryos with 69.4% weight in RIF cohort (Tan et al., 2018; Cozzolino et al., 2020). This may partly explain the above differences because RIF patients most likely to benefit from a greater proportion of euploid embryo-transfer with pET guided by ERA. In addition, the current study has the intrinsic limitation of being a meta-analysis, for instance, different ethnic groups and different embryo transfer protocol, and so on. Well-designed clinical studies are warranted to verify these findings.

In addition, several novel prediction tools [ER Map^®^/ER Grade^®^(Enciso et al., 2018), Win-Test (Haouzi et al., 2021), rsERT (He et al., 2021) and so on] for endometrial receptive have been developed to determinate the displaced WOI to guide the pET in recent experiences, which proved that pET can significantly enhance pregnancy outcome in patients with RIF. However, they are still in the initial stage, and their clinical value needs to be further verified.

### Limitations

There were some unavoidable limitations in this study. Firstly, our results are partly limited by the small number of enrolled patients, and heterogeneity in characteristics of subjects (including IVF cycles protocol, number of transferred embryos, days for ET [cleavage-stage vs. blastocyst-stage embryos]), and poor methodological quality of original studies. Besides the inconsistent use of endometrial biopsy protocol (natural or HRT cycle), as well as the age and BMI of patients, may constitute additional confounding factors in estimation of the impacts of ERA test on IVF outcome. Thirdly, the interval of the first ERA biopsy to the first pET varied among articles, potentially generating bias in consideration of the impact of endometrial biopsy (scratching) on pregnancy rate. Finally, the status of every embryo (euploidy) was not confirmed in some studies, which did not exclude embryo aneuploidy as cause for implantation failure. Moreover, the invasive nature of the test, the requirement of embryo vitrification and economic cost constitute some of its limitations (Ben Rafael, 2021). Hence, the present meta-analysis demonstrates that the value of ERA may also need to be interpreted with caution.

## Conclusion

The current systematic review and meta-analysis reveal that considering the about one third of infertile women could suffered from displaced WOI, the ERA test emerged as a promising tool. Although the present meta-analysis demonstrates that patients with general good-prognosis may not benefit from ERA, pET guided by ERA significantly increases the chances of pregnancy for non-receptive patients with RIF of endometrial origin. Regarding the small number of published literatures and the significant heterogeneity among studies, there is a need for more high-quality prospective randomized controlled trials to confirm the clinical value of ERA for different populations.

## Data Availability

The original contributions presented in the study are included in the article/Supplementary Material, further inquiries can be directed to the corresponding author.
